# Wnt pathway activation functionally reprograms human antigen-specific T cells

**DOI:** 10.1186/2051-1426-2-S3-P4

**Published:** 2014-11-06

**Authors:** Yen-Ling Chiu, Bo-yi Sung, Catherine Bessell, Mathias Oelke, Jonathan Schneck

**Affiliations:** 1 Department of Medicine, Far-Eastern Memorial Hospital, New Taipei City, Taiwan, Taiwan; 2 Department of Pathology Johns Hopkins University School of Medicine, Baltimore, Maryland, USA; 3 Department of Medicine, and Institute for Cell Engineering, Johns Hopkins University School of Medicine, Baltimore, Maryland, USA; 4 Johns Hopkins School of Medicine, Department of Pathology, Institute for Cell Engineering, Baltimore, Maryland, USA

## 

Polyfunctionality is a hallmark of protective immunity, yet the molecular mechanisms governing polyfunctional T cells are poorly understood. After TCR activation, naïve CD8^+ ^T cells undergo proliferation and differentiation, which lead to effector functions and memory subset development. However only a portion of activated T cells develop into memory CD8^+ ^T cells and with chronic stimulation become terminally differentiated and exhausted CD8^+ ^T cells, as defined by CCR7^-^/CD45RA^+^, and functionally impair effective immune responses [1]. We therefore probed the ability to reverse terminally differentiated antigen-specific cells using pharmacological agents. Stimulating human memory CD8^+ ^T cells with cognate TCR stimulation in the presence of Wnt agonist enhances polyfunctionality and stemness. Both M1-influenza^+ ^and CMV^+ ^CD8^+ ^T cell responses were reprogrammed and revealed sustained effects from initial Wnt pathway activation *in vitro*. Future work with cancer antigens and reprogramming of differentiated CD8^+ ^responses could lead to improved *in vitro *culture conditions for adoptive immunotherapy.
